# Reflections in focus: a qualitative Photovoice-informed study on pediatric patient and caregiver hospital experiences

**DOI:** 10.1186/s12887-025-06281-5

**Published:** 2026-01-09

**Authors:** Freja K. Ekman, Niisoja M. Torto, Katherine Hu, Catharine Bowman, Olubunmi A. Fariyike, Justin J. Yates, Syamantak Payra, Charbel Bou Khalil, Ezra T. Yoseph, Rowan M. Ings, Ruby E. Reed, Samuel T. Rodriguez

**Affiliations:** 1 Stanford University School of Medicine, Stanford, CA USA; 2 Stanford University, Stanford, CA USA; 3 Department of Anesthesiology, Perioperative, and Pain Medicine, Division of Pediatric Anesthesiology, Stanford School of Medicine, Stanford, CA USA

**Keywords:** Pediatrics, Medical Humanities, Photovoice, Caregiver, Narrative Medicine

## Abstract

**Background:**

Pediatric patients and caregivers face numerous challenges throughout their healthcare journey, often experiencing feelings of lost agency and lost voice. Photovoice, a participatory methodology combining photography with storytelling, empowers participants to share their experiences from their own perspective. However, its use in pediatric hospitalizations has been limited. Using Photovoice-informed methods, this study examines how pediatric patients and caregivers experience illness and navigate the healthcare system, helping identify opportunities to improve care.

**Methods:**

This study was conducted at Lucile Packard Children’s Hospital between July 2022 and April 2024. Pediatric patients and adult caregivers were recruited from inpatient and hospital-based outpatient settings through the Child Life department. Participants captured photographs of their hospital experiences using personal or study-provided cameras. Semi-structured interviews were conducted with participants to explore the meaning behind their photographs. Team-based inductive thematic analysis of the interviews was then conducted to synthesize care recommendations.

**Results:**

Eight pediatric patients and six adult caregivers participated. Five themes emerged: (1) life before vs. currently vs. after; (2) environment; (3) playing and being a normal kid; (4) joy, gratitude, and appreciating small things; and (5) resilience and mental health. Patients and caregivers described distinct yet complementary perspectives. Children focused primarily on immediate experiences and processing current circumstances, whereas caregivers emphasized broader responsibilities and long-term implications. Participants’ interactions with the hospital environment particularly highlighted these differences. Patients found symbolic meaning in physical objects, while caregivers focused on interpersonal dynamics. Both groups valued the moments depicted in their photos as reminders of strength and progress, though caregivers noted the emotional complexity of certain images representing both suffering and hope. While both groups emphasized the importance of play and normal routines, patients viewed these as enjoyable distractions while caregivers saw them as evidence of healing. Despite these differences, gratitude for ordinary objects and interpersonal support emerged as universal sentiments.

**Conclusion:**

This study highlights the unique experiences of pediatric patients and adult caregivers in navigating illness and prolonged hospitalization. Our findings emphasize the importance of Photovoice methodology in capturing the nuance of the patient and caregiver experiences as well as further opportunities to improve pediatric care.

**Supplementary Information:**

The online version contains supplementary material available at 10.1186/s12887-025-06281-5.

## Introduction

Hospitalization is often a disruptive and emotionally challenging experience for pediatric patients and their families. For children, hospital admissions often remove them from familiar routines and social environments, leading to a limited sense of autonomy and increased exposure to unfamiliar, sometimes intimidating, medical settings. These disruptions can ultimately have a lasting impact on pediatric patients’ emotional well-being and social development [1]. Specifically, a recent scoping review revealed that pediatric patients discharged from the pediatric intensive care unit (PICU) experience rates of post-traumatic stress disorder (PTSD) as high as 85%, with symptoms lasting months to years after hospitalization [2]. Notably, compared to nonhospitalized children, such patients have also been found to have lower intelligence quotient (IQ) scores at two-year follow-up as well as a higher prevalence of behavioral and emotional problems at four-year follow-up, highlighting the long-term negative psychosocial effects of hospitalization in children [3]. Conversely, the caregivers of these patients can also experience emotional distress, and they must often balance the roles of patient advocate and emotional support provider for their child during their hospital stay [4]. This has been shown to have a direct, negative impact on the mental wellbeing of caregivers, leading to severe anxiety, major depression, and decisional conflict [5]. Further, beyond the detrimental impacts on individuals, such acute hospitalization events have also been shown to harm the relational dynamics of the family unit, leading to impaired social functioning [6, 7], strain in relationships with nonhospitalized siblings [8], and reduced ability for families to adapt to future challenges [6]. These effects may last for years [1]. Moreover, pediatric patients and their adult caregivers have been shown to display similarly high rates of PTSD related to hospitalization [9]. Importantly, parents who did not experience PTSD symptoms were more likely to have children who did not experience PTSD as well, suggesting an important role for family relationships in mitigating the negative psychological effects of hospitalization [9].

Taken together, acute hospitalization portends a high risk of negative impacts to individual mental health and overall family dynamics even long after discharge, which may ultimately impact recovery and shape how families remember their healthcare experience.

While existing studies have provided valuable insight into the emotional burden and coping mechanisms associated with pediatric hospitalization, much of this work relies on clinician-reported outcomes or caregiver/proxy reports that may not fully capture the child’s own perspective.

Photovoice offers a unique participatory approach that enables patients and families to document and reflect on their lived experiences through their own words and images [10]. This method has been shown to not only promote self-expression, but also provide healthcare workers with patient-focused insights [11, 12]. In pediatric settings, Photovoice has been used to explore diverse topics, including chronic disease and chronic pain management and promotion of lifestyle changes [13–20], but its application within acute settings, such as inpatient hospitalization, is limited. Of the studies focused on the hospital experience, only three explored the perspective of the hospitalized pediatric patient, each focusing on a specific aspect of hospitalization, such as hope or fostering mental wellbeing [14, 15, 18]. Therefore, the potential for Photovoice to capture everyday realities in an unbiased fashion, capturing both positive and negative aspects of the pediatric hospitalization experience for patients and their caregivers alike, remains relatively underexplored.

In this work, we leverage a Photovoice-informed approach to investigate how pediatric patients and their caregivers experience the hospital environment. While many participants in this study live with chronic medical conditions, this specific study is centered less on the experience of long-term illness which has been explored previously. Instead, we examine how pediatric patients and caregivers perceive and navigate the hospital environment with the goal of offering insights into how prolonged hospitalization impacts family dynamics, child development, and psychosocial well-being.

## Methods

### Study conceptualization

This qualitative observational study is informed by Photovoice, an innovative participatory action research methodology. Participatory action research (PAR) is a collaborative approach that engages participants as active contributors throughout all stages of the research process. First described by Wang and Burris (1997), Photovoice, rooted in PAR, uses photography to empower individuals to document and reflect on their lived experiences, typically in a group setting [21]. Participants take photos, engage in collective discussion, guide theme generation, and often participate in public exhibitions or dissemination of findings as a form of advocacy [21, 22]. Our study is Photovoice-informed and adapts Photovoice to a clinical setting. While remaining grounded in the core principles of Photovoice—such as, participant empowerment, visual expression, and participant-driven storytelling—we made intentional changes to address the unique challenges of the hospital setting, including patient privacy and logistical constraints. Similar Photovoice-informed methods have been used in prior clinical studies as a way to elevate patient voices while accommodating the constraints of healthcare environments [23, 24].

While our approach shares some features with photo-elicitation—such as using photographs in one-on-one interviews [25]—it aligns more closely with Photovoice. We chose Photovoice to inform our study due to the participant-centered nature of the methodology and its emphasis on participants generating their own photos and sharing their own narratives [26]. In contrast, photo-elicitation often involves researcher-selected images and functions primarily as an interview tool rather than a participant-driven process [26].

In line with Photovoice principles, this study featured pediatric patients and their caregivers capturing photographs reflecting their experiences and engaging in semi-structured interviews to share the meaning behind these images. The project culminated in public gallery installations where participant photos and excerpts were displayed and patients and caregivers were invited to share their stories more directly with the audience. However, we intentionally deviated from traditional Photovoice elements such as group discussions and collaborative theme generation. Given the clinical, developmental, and emotional needs of our pediatric population, as well as logistical challenges in the hospital environment, all interviews were conducted individually. Group-based reflection was not feasible and could have compromised confidentiality and participant comfort. As a result, researchers synthesized themes across participants based on their photographs and narratives—an adaptation that preserved the importance of the participant voice in Photovoice while ensuring ethical and practical feasibility.

### Recruitment procedures

Pediatric patients and their adult caregivers in this study were recruited at Lucile Packard Children’s Hospital (LPCH), an academic tertiary care children’s hospital in Palo Alto, California. Patients and caregivers were recruited through Child Life specialists. As specialists in child care and well-being, Child Life specialists are consulted by a patient’s clinical team to help patients and families navigate the hospital stay and provide care enrichment and wellness activities throughout the patients’ hospital course.

Eligible pediatric patients were at least 4 years of age (and up to 18 years of age), an age cut-off that was selected in consultation with Child Life specialists, based on developmental readiness for meaningful participation in the project. Pediatric patients over the age of 18 were also included if a Child Life specialist assessed their developmental age to be under 18 and deemed them appropriate for the study. When caregivers were the primary participants, there were no age restrictions placed on their children, who ranged from newborns to adolescents. Recruitment of patients and caregivers occurred across all inpatient units and hospital-based outpatient clinics, including general pediatrics, surgery, oncology, cardiology, dialysis, and labor and delivery, among others. Outpatient clinics were included if they were physically based within the hospital and served patients whose experiences involved frequent or prolonged interactions with the hospital system, such as dialysis. This approach allowed us to more fully explore how the impact of pediatric hospitalization extends beyond inpatient stays, shaping the experiences of children and caregivers who engage in frequent, hospital-based outpatient care. Specific study inclusion and exclusion criteria included in Table 1.

**Table 1:** Inclusion and exclusion criteria

Inclusion Criteria	Exclusion Criteria
● Pediatric patient at Lucile Packard Children’s Hospital in the inpatient unit or hospital-based outpatient unit ● At least 4 years old ● Able to speak and understand English ● Adult caregiver or parent (aged 18 or older) of pediatric patient meeting the above criteria	● Caregiver or parent that does not consent for both their child and themselves ● Pediatric patients that do not assent to participation ● Pediatric patients whose care team identifies their medical needs as too severe to participate, or for whom participation would complicate their care plans ● Participants with a history of epilepsy that self-report to the research team that camera flashes may be triggering to them

Participants were identified through purposive sampling, with the intention of using a heterogeneous sampling approach to promote the diversity of perspectives on pediatric hospitalization. To support this, Child Life specialists were asked by our team to help identify participants that fit the inclusion criteria from across various hospital units and pediatric patients of different ages. Child Life specialists, in collaboration with the care team, assessed whether pediatric patients and their caregivers were clinically and socially appropriate for participation—meaning they were likely to enjoy and meaningfully engage in the project based on their emotional readiness, communication abilities, and overall clinical status. Researchers then met with potential participants in-person to explain the project and, if there was patient and caregiver interest, obtain informed consent.

### Human subjects approval

This study and all associated consent forms were approved by the Institutional Review Board of Stanford University (IRB 64507).

### Data collection

Consented pediatric patients and adult caregivers were asked to take photos documenting their experiences with illness and navigating the healthcare system. Pediatric participants received prompts such as: *“Take a photo of something that captures your experience living with your condition.”* Caregivers were given parallel prompts, including: *“Take a photo of what it’s like to care for your child in the hospital.”* A complete list of prompts is available in Supplemental Material 1.

Although participants were encouraged to avoid photographing identifiable individuals, they were permitted to do so if proper consent was obtained. As part of the consent process, pediatric participants and their caregivers signed a media-release form that allowed photos to be taken of themselves. They were also counseled on the ethics of photo-taking and the importance of obtaining consent before photographing others. Participants were given additional media-release forms in case they wished to include other people in their photos, such as family members or healthcare staff. Only photographs with proper consent were included in the final dataset for analysis.

Participants were given the opportunity to take photos with either (1) a camera provided by the research team that simultaneously printed instant film photos and saved digital copies (Kodak STEP Instant Print Digital Camera) or (2) their personal cellular device, depending on their preference. Participants were given a flexible timeframe ranging from one week to up to twelve weeks to document their experiences during hospitalization. Recognizing the unpredictable realities of the hospital setting and participants’ changing clinical statuses, we worked individually with each participant to determine the time they needed for documentation. Participants were contacted weekly or biweekly to assess their interest in continuing or concluding the process. This participant-driven approach to determine the documentation duration reflects the principles of participatory research [27]. While the length of the documentation period and the total number of photos taken varied across participants, the number of photos patients were asked to select for interview was standardized to a subset of three to five per participant.

Photographs were stored securely on a HIPAA-compliant, Stanford-affiliated Box server (Box, Redwood City, CA). Participants who used study-provided cameras kept their instant film photos, while digital versions were uploaded by the research team. Participants using personal devices submitted their selected photos through a secure online form, which uploaded directly to the encrypted Box server. After the documentation process, semi-structured interviews were conducted with participants. Interviews were held either in-person or virtually based on participant preference and scheduling needs and typically lasted between 30 min and one hour. Virtual interviews were done through the Zoom platform (Zoom Communications, San Jose, CA). Sessions were conducted by trained members of the research team at a mutually agreed-upon time and took place in a private and distraction-free setting when possible. Pediatric participants were interviewed with caregiver support as needed, particularly for younger children. Interviewers adapted their language, tone, and pacing in real time based on the child’s developmental stage and level of engagement. Caregiver interviews used language and framing appropriate for adult participants.

For these interviews, all participants were asked to identify three to five photos that most captured their experiences. While Wang et al. recommends selecting one to two photos in traditional adult group-based Photovoice, we expanded this number to better support pediatric participants in an individual interview format [28]. A greater number of photos provided more prompts for reflection and helped maintain engagement, particularly for younger participants. This adaptation aligns with other Photovoice-informed studies featuring children and adolescents, where participants have been encouraged to share multiple photos to accommodate youth communication styles [29–31]. The research team reviewed the chosen images with participants during the interview and excluded any images with identifiable individuals that had not signed consent or media-release forms from analysis and dissemination.

Participants were then asked open-ended questions about what their photos represented (see interview guide in Supplemental Material 2). These questions, which were used for both pediatric patients and caregivers, were adapted from the SHOWeD technique described by Wang et al. [28], with modifications to simplify language for pediatric participants and tailor the prompts to the hospital context [9]. These interviews were recorded and transcribed using otter.ai (Otter AI, Mountain View, CA USA) and manually checked for accuracy by the research team in preparation for analysis.

### Data analysis

Team-based inductive thematic analysis was used to analyze the participants’ interviews [32]. This qualitative approach was adopted to allow key themes and insights to be discovered organically from the data itself and ensure that participants’ experiences could be shared and analyzed without preconceived notions or biases [33]. The analysis was conducted after all interviews were completed to allow for a comprehensive synthesis of the data. Thematic saturation was not formally monitored, given the small sample size and our intention to ensure that all participants had the opportunity to share their unique perspectives [34].

The data was analyzed using Dedoose (Version 10.0.25, SocioCultural Research, Los Angeles, CA USA). Three researchers reviewed all the transcripts and independently identified recurring themes and patterns [35]. This initial phase yielded nearly 40 unique sub-themes, which were discussed, refined, and organized into a structured coding framework consisting of 5 main themes, each with corresponding sub-themes [35]. To validate this framework, the researchers independently coded two new transcripts using the developed framework on both pediatric and adult caregiver transcripts [36]. The pilot phase also served to standardize the coding methodology and ensure consistency among researchers [36]. Based on this pilot phase, refinements were made as needed before proceeding to full transcript coding [36].

For the analysis, one researcher coded all of the transcripts, which were secondarily coded by an alternate researcher, ensuring that every transcript was reviewed independently by two people [36]. A code reconciliation process followed, where discrepancies were discussed and resolved to ensure cohesiveness for the final analysis [36].

Once coding was complete, the coded transcripts were analyzed by theme [33]. Within each theme, researchers identified representative quotes, examined common patterns and ideas, and compared perspectives between pediatric patients and adult caregivers. This process provided a thorough understanding of shared and divergent experiences, offering deeper insight into the themes that emerged from the data.

## Results

### Participants

Fourteen participants were interviewed. Eight participants were pediatric patients and six were caregivers. Demographic information of these participants is described in Table 2. All patient and caregiver participants were female despite enrollment being open to any gender. However, some female adult caregivers spoke about taking care of male pediatric patients. Similarly, while all caregiver–child dyads were mother–child pairs, enrollment was open to any type of caregiver relationship. The reasons for non-participation were not obtained from individuals who declined to participate.

**Table 2:** Participant demographics

Characteristic	Category	Number of patients (*n* = 14)
*Category of participant*	Pediatric patient alone	5
	Adult caregiver alone	3
	Pediatric patient and adult caregiver from same family	3
*Gender of participant*	Female	14
	Male	0
*Gender of child of adult caregiver participants*	Female	4
	Male	2
*Age of participant*	Age of pediatric patient participants	11.75 +/- 4.83 years (range 5–20 years)
	Age of pediatric patient associated with adult caregiver	4.83 +/- 6.62 years (range newborn-13 years)
Hospital unit of pediatric patient*	Dialysis	1
	Cardiology	6
	General Surgery	1
	Oncology	2
	Neonatal Intensive Care	1
	General Pediatrics	1

*pediatric patient and adult caregiver from the same family were only represented once in this quantification

### Key themes in patient and caregiver narratives

The following five themes were identified when analyzing participant interviews: (1) life before vs. currently vs. after; (2) environment (3), play and being a normal kid (4), joy, gratitude and appreciating small things, and (5) resilience and mental health. Theme definitions and illustrative quotes are presented in Table 3. Additional representative quotations can be found in Supplemental Material 3. Representative quotes in the main text are labeled as *Adult Caregiver of [patient age]* to indicate an adult caregiver participant, where the number reflects the age of their hospitalized child (e.g., *Adult Caregiver of 3-year-old patient*), and as *Pediatric Patient*,* [patient age]* to indicate a pediatric participant, where the number represents the patient’s own age (e.g., *Pediatric Patient*,* 7 years old*).

**Table 3:** Representative quotes across all five identified themes. Examples provided for both patient and caregiver participants

Theme	Proposed Definition of Theme	Illustrative Quotes
1: *Life Before vs. Current vs. After*	Reflects on past, present, and future experiences, capturing changes and uncertainty as circumstances evolve over time	“It could tell people that you even though you start off doing something that you don’t want to do, doesn’t mean you have to do it forever.” – 11 year old patient “Seeing like my daughter go through all these like drastic changes, like having to get a heart transplant. Like, it just, it just changed instantly, like we were at home, you know, living normal lives. And then now we’re here in the hospital and don’t know when we’re gonna go home, you know, and she’s kind of like bound to the bed” – Caregiver of 1 year old patient
2: *Environment*	Involves interactions with and interpretations of physical surroundings and the meaning derived from those surroundings	“This pole was the thing that was keeping me out of a lot of things that I probably would have wanted to be in. After surgery, well like the very first surgery that I had, that pole was filled with machines, a lot of, you know, medicines, antibiotics, and all the things that I needed to keep me alive. Well, there’s a huge change, as you can see, that there is nothing there anymore, that there’s only a feeding tube that is hanging on for dear life. And, you know, it’s a huge reminder of how it was before and how it is now. Because that means I made it, like, I don’t need it anymore” – 15 year old patient “Having a lot of empathy for the patients and the caregivers, and being able to look at it and realize how on the outside I was and my husband was like we. You know this is our like newborn child, and you can just tell from this picture like we were far away.” – Caregiver of newborn
3: *Playing and Being a Normal Kid*	Captures perceptions of childhood play and normalcy and how experiences align with or differ from it	“Even though you’re in the hospital it can still you can still have fun” – 20 year old Pediatric Patient “[She’s] had three strokes. So all the therapy and interaction helps with her rehabilitation process. So this is where she’s the happiest, playing with toys. [Her] Child Life [specialist] helps her stand up, play with toys, you know, get her interacting. And this is why I picked this photo, because it’s like, a strong part of her journey” – Caregiver of 1 year old patient
4: *Joy*,* Gratitude*,* and Appreciating Small Things*	Highlights moments of love, comfort, and appreciation, and the recognition of things often taken for granted	“This photo is of me and my mom when we first got the camera. We wanted to take the picture because my mom is always with me … Always love your mom” – 14 year old patient “Even in the worst experience, you could always find some sort of joy and something that makes you smile” – Caregiver of 5 year old patient
5: *Resilience and Mental Health*	Demonstrates strength, growth, and self-advocacy along the journey to overcome obstacles, embrace support, and pursue goals with hope and determination	“I took this photo because I can look back at it and I can think, oh well like, if I’m going through anything really bad. I can just look back at the photo. And think of the like more rough times that I’ve been through in the past” −15 year old patient “We bring color to our life, and we don’t let what’s happening, you know, put us in a dark place, because we keep the light shining, and the colors bright” – Caregiver of 13 year old patient

#### Theme 1: life before vs. currently vs. after

**Fig. 1:**
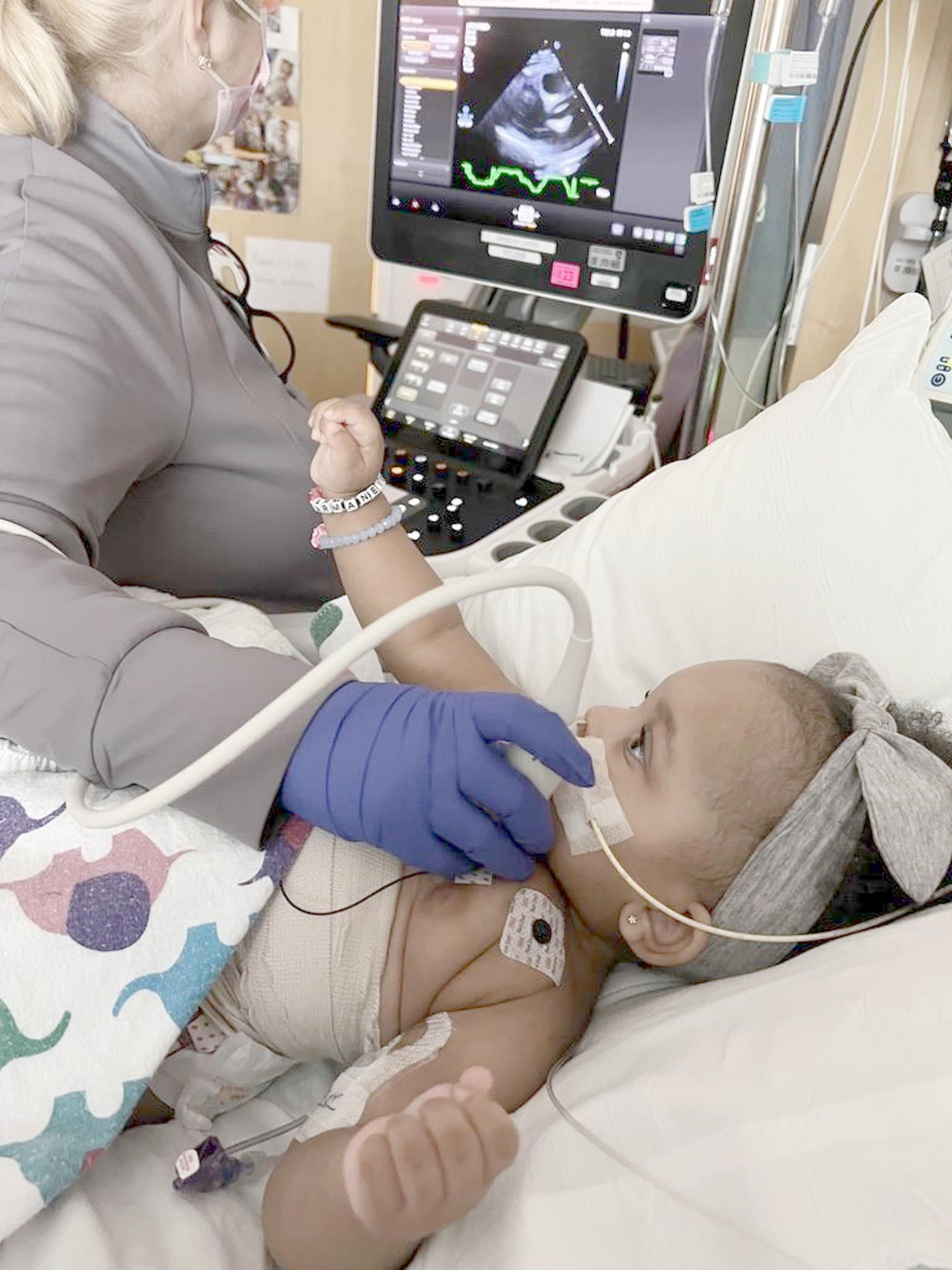
Photograph by adult caregiver of child getting echocardiogram

In interviews, caregivers frequently highlighted the dramatic contrast between their previous healthy lives and their current hospitalization. The vivid contrast with their recent “normal” life intensified the hospital experience for many caregivers, evidenced by one caregiver who stated, “It just changed instantly, like we were at home, you know, living normal lives. And then now we’re here in the hospital and don’t know when we’re [going to] go home, you know, and she’s kind of like bound to the bed” (Adult Caregiver of 1 year-old patient) when describing a photo of her child receiving an echocardiogram **(** Fig. 1**)**. Caregivers further detailed how their world perspective significantly narrowed upon entering the hospital as their attention became centered on their child’s medical care. One caregiver described, “there is this whole other kind of world of events happening outside of the hospital and for me and my experience it was just like I was physically still recovering and having to walk back and forth between the postpartum unit and the ICU was really hard and a little bit isolating” (Adult Caregiver of 2 month-old patient).

Both caregivers and patients primarily reflected on these temporal changes with hope, motivation and inspiration. For example, one pediatric patient reflected on a photo she took of photos from home displayed on her room door, saying,

“So all of those pictures are memories of before I came here and how my life was, you know, me growing up, being with my family. Most of them were really very special moments that I had in my life. So kind of just looking at having that on my door. It’s really nice to see because it kind of shows me how far I’ve come and that I can get that life that may not be the same when I come back. But I’ll definitely have like, a lot of lessons learned after so that way I can, you know, grow to have my old self back but not entirely” (Pediatric Patient, 15 years old).

A caregiver similarly reflected,

“I guess it’s just nice to have her up and moving. There was so so long that she had to just be in the bed, which is really hard to see. And for a six year old. And you kind of see their muscles kind of like go away and just get really scrawny and skinny. So it was nice to just be like, able to see her up and like getting some strength back” (Adult Caregiver of 6 year-old patient).

Caregivers also tended to focus on feelings of fear and sadness associated with these changes in time. For instance, one caregiver shared,

“my birth experience with him was really like pretty positive…and the hours after he was born were the same. And so I think this picture really represents a very different turn of events. It was so scary because he was so tiny, and I didn’t know what was happening, and I wasn’t very empowered, and I just wanted to be able to hold him. He is such a tiny baby” (Adult Caregiver of 2-month old patient).

In line with this hope, pediatric patients often tended to emphasize how the differences between their normal life and hospital life could be used as a helpful reminder to their future selves that difficult situations are temporary, not permanent. For example, one patient expressed, “[This picture] could tell people that, even though you start off doing something that you don’t want to do, doesn’t mean you have to do it forever” (Pediatric Patient of 11 year-old patient). Caregivers also described a gradual adjustment to their new circumstances, with one describing, “Every month, she gets an Echo to see like, if her heart is shrinking, if it’s still the same size… at first, they used to, like, stress me out a little bit because she would like be going like crazy. But now she’s like, just relaxed. So they don’t really bother me because we’ve been here for so long. So it’s normal at this point” (Adult Caregiver of a 1 year-old patient).

#### Theme 2: environment

**Fig. 2:**
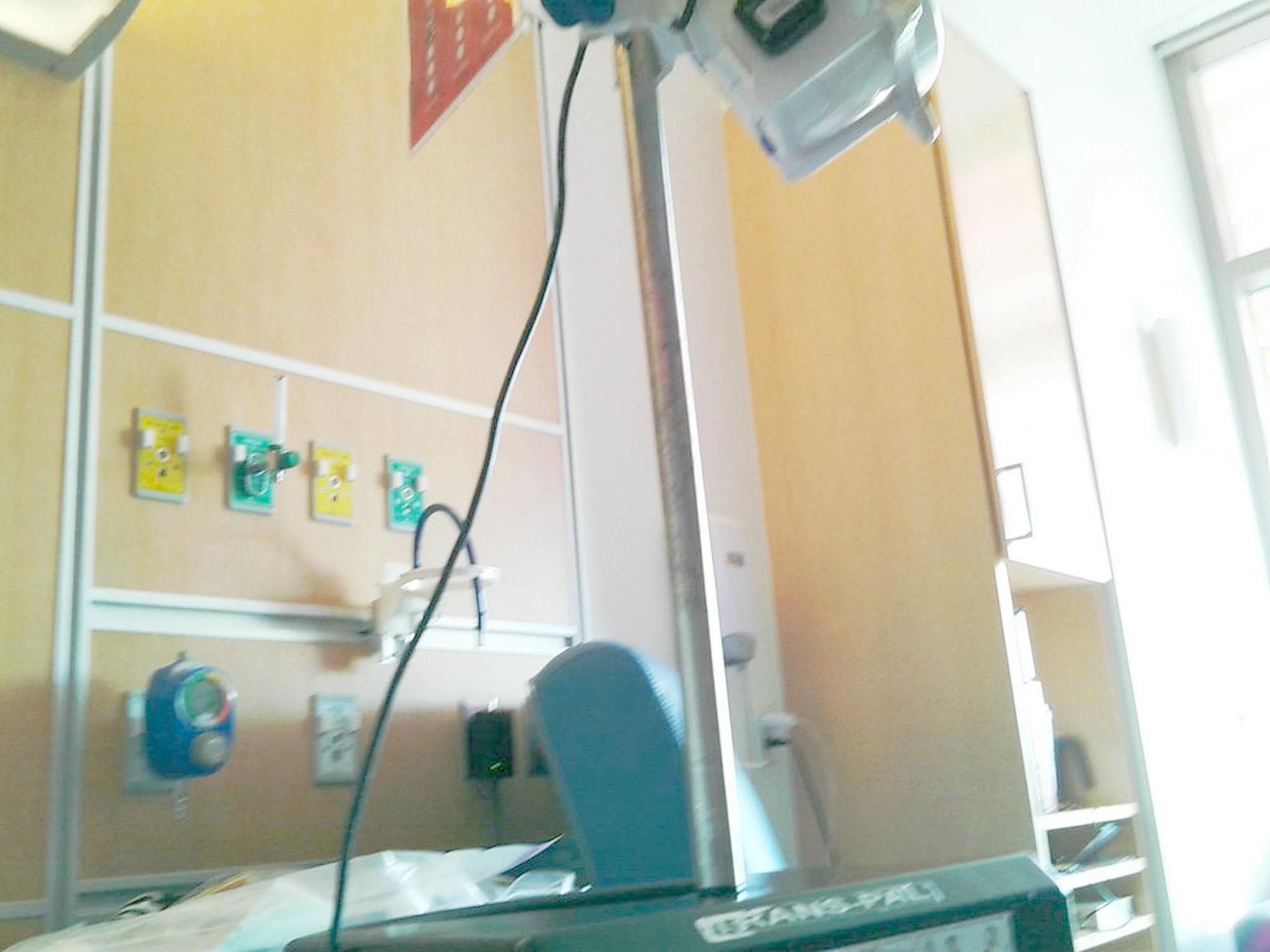
Photograph of IV pole taken by pediatric patient towards the end of her hospital stay

While both pediatric patients and their caregivers discussed their physical surroundings, they interpreted their environment differently. Patients took pride in personalized room decorations and often found symbolic meaning in physical objects. One patient shared, “I had a lot of nature Legos like flowers, and I did have a cactus and succulent garden. And a bonsai tree. And I also had a Lego aquarium. And I got bored of my room looking so boring. So I used like, streamers and gold stars, and I hung them” (Pediatric Patient, 11 years old). Patients valued mementos from loved ones as bridges to the outside world. One patient shared, “And the day before my aunt, my uncle and my cousins left, they actually put all of these photos up. And they drew [a] little something for me. So that way I can look up to like, since they’re not here…so [with these photos] they’re basically always here”(Pediatric Patient, 15 years old). This patient continued to share how pre-hospitalization photos were inspiring as they “[show her] how far [she’s] come and that [she] can get that life back” (Pediatric Patient, 15 years old). Of particular note, several patients derived symbolic meaning from photographs they had captured of medical equipment and rehabilitation tools used during their hospital stay. For example, one patient (age 15) describes a photo taken of her IV pole (Fig. 2):

​“This pole was the thing that was keeping me out of a lot of things that I probably would have wanted to be in. After surgery, … that pole was filled with machines …, medicines, antibiotics, and all the things that I needed to keep me alive. Well, there’s a huge change, as you can see, that there is nothing there anymore, that there’s only a feeding tube that is hanging on for dear life. And, you know, it’s a huge reminder of how it was before and how it is now. Because that means I made it, like, I don’t need it anymore” (Pediatric Patient, 15 years old).

Caregivers, on the other hand, focused more on spatial and interpersonal relationships within the hospital environment. Several caregivers highlighted the emotional difficulty of physical separation from their child during their child’s hospitalization. One caregiver described,

“I don’t like not being here at the hospital, I get a lot of anxiety. I always worry that someone’s gonna come in and want to talk to me and like, my husband is usually here, but he went home for like a week and a half in the middle to work. And so it stresses me out not to be here, but also they’re so young, and may need me and so it was really hard, because so when I’m there [with my other children], I feel like I should be here” (Adult Caregiver of 6 year-old patient).

**Fig. 3:**
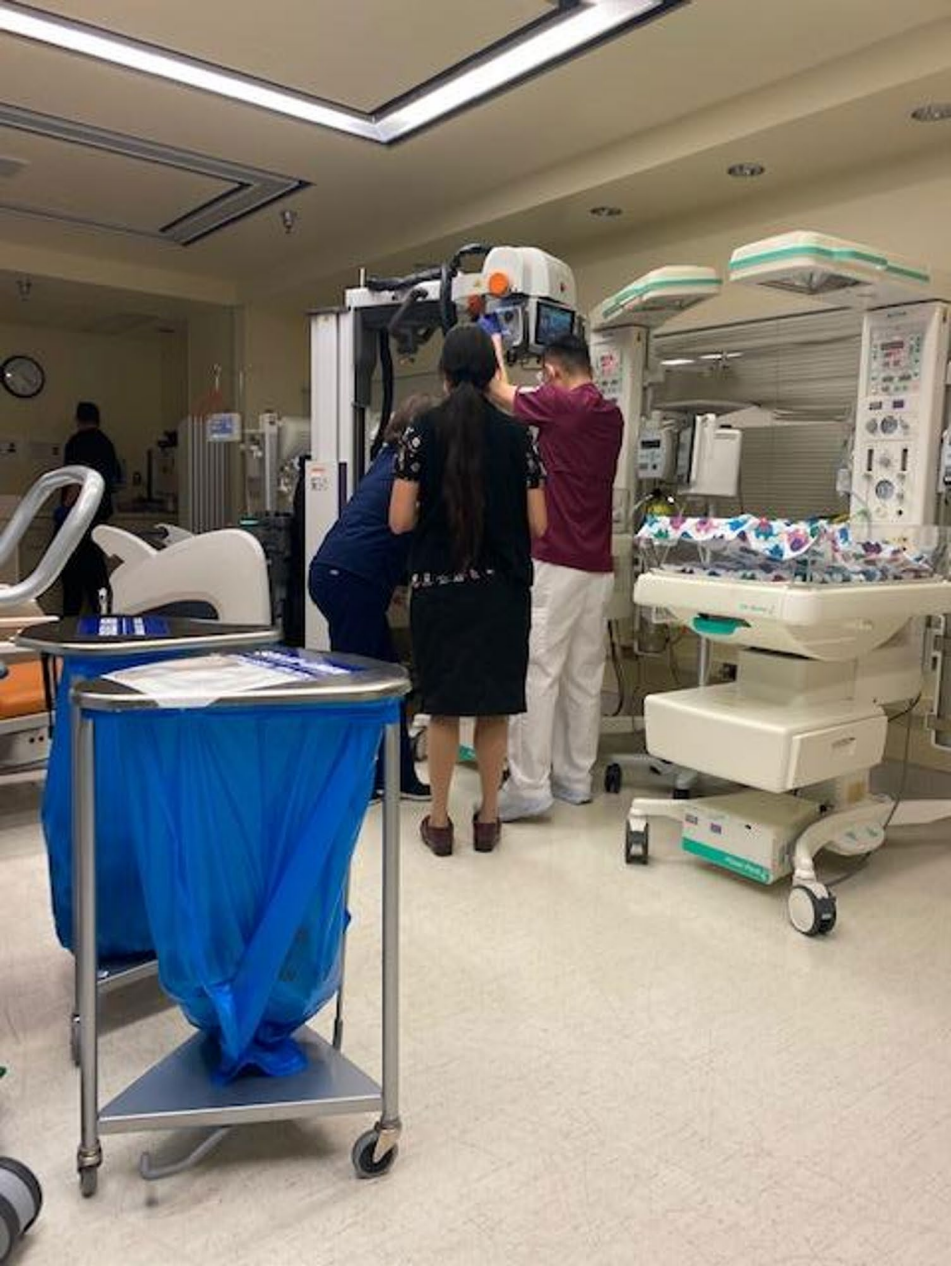
Photograph by adult caregiver observing the care team surrounding her newborn child

This sense of separation extended beyond simply being away from the hospital; it also included feeling disconnected even while in the same room. For example, one caregiver of a child in the NICU noted while her child was being evaluated by the care team and she was standing at the edge of the room, “You know, this is our newborn child, and you can just tell from this picture like we were far away” (Adult Caregiver of 2 month-old patient) (Fig. 3).

One participant emphasized how clear, compassionate communication from healthcare providers can ease the emotional toll of being physically separated from their child, recalling a particularly impactful moment:

“It was like a physical barrier between us and our baby, so I think the lesson is like, have as much empathy as possible. Do more to explain to the parents like what’s happening, and how the outlook is. I will say this is actually really important. The nurse in black was amazing, and she saw me crying like after [my child] was settled, and she like stood face to face with me and held my hands and said: ‘You know this is really scary. They are just finding out you know what’s wrong, so we can help him. It’s okay, like he’s breathing. I know this is scary,’ but like she just was really really reassuring. And so I think that was a lesson, too, for you know, nurses and doctors and other hospital workers, to really take that moment when the immediate crisis is over, to look someone in the eye and tell them like this is what’s happening” (Adult Caregiver of 2 month-old patient).

Finally, a consistent theme across both patient and caregiver interviews was the perceived divide between the hospital environment and the outside world. Both groups expressed that they found the hospital’s sterility emotionally challenging but noted that incorporating living elements like plants helped to ease these feelings. One patient noted, “I don’t really know if it helps the hospital or other people, but I know I like seeing plants you know, alive and everything if they keep watering them” (Patient Participant, 20 years old). A caregiver also shared, “we miss [the] outdoor[s] [while] stuck in here. Unfortunately, we can’t go outside… I’m going a little crazy because we have been in this room for four weeks I think…the view is helpful to look at the nice view but the sad part is I can’t go out” (Adult Caregiver of 4 year-old patient). Caregivers observed that in-hospital play areas helped children cope with these feelings of confinement, with one noting, “they have a lot of like activities for the kids to do, which helps a lot. You know, like they have physical therapy, speech therapy, music therapy. They have the librarian. You know, it’s like a lot for them to do so they’re not just sitting in here, they’re developing” (Adult Caregiver of 1 year-old patient). However, both groups identified access to the outdoors as their most effective coping mechanism. One pediatric patient described gaining significant strength from her connection to nature during visits to a cactus garden near the hospital:

*“*The last [picture] is a really cool cactus tree that was trying to lean towards the sun and where it usually is. And I just thought it was really cool. It has tons of spikes along the trunk. And it made me think how brave it was and how it kind of was like me… It was surviving its life, trying to survive its life” (Pediatric Patient, 11 years old).

Also emphasizing the importance of outdoors, one caregiver shared,

“I feel like if she could get out more like outside, like, you know, just breathing fresh air, she hasn’t been outside since like, September. So just getting outside and like, off to the hospital floor down in the cafeteria or to just around people and [I] think that will help a lot” (Adult Caregiver of 1 year-old patient).

#### Theme 3: play and being a normal kid

Both children and caregivers described seeking as much normalcy as possible in the hospital. Children expressed desires to both play and be treated like typical children. One patient shared, “even though you’re in the hospital, you can still have fun” (Patient Participant, 20 years old). Older patients particularly noted the value of staff using humor to tease and treating them in an age appropriate manner. This same patient described one memorable moment saying “Well, [my nurse] was funny. Everybody made a joke how she acted like my mother. And she’s like, ‘Well, my daughters are her age, though.’ And just the way you know, she treated me and she did a lot” (Patient Participant, 20 years old). Patients also described finding comfort in ordinary routines, such as family meals at cafeteria tables, with one sharing, “Well, since we’re at this hospital, every day [my family] will come and they get to talk and play. They stay all day for lunch or sometimes dinner. They usually stay for dinner. One time we went downstairs and ate together as a family” (Pediatric Patient, 5 years old). These activities provided hope for a full recovery in the future. For example, capturing her resilience and her desire for normalcy, one patient explained “Well, I watched the cooking channel, not only because I want to get better at cooking, but because, you know, I also have a lot of faith that I’m going to eat something like that someday, again” (Pediatric Patient, 15 years old). These activities were a source of distraction, as one patient noted “one of the things that keeps me occupied while I’m here is just having fun and doing things. So I’m not always just worried about what’s going on with me” (Pediatric Patient, 12 years old). While some patient photographs prompted thoughtful reflection, others captured moments of simple amusement and normal play. One patient simply described “I took this picture because it looks funny to me because there’s a dog on my head” (Pediatric Patient, 14 years old).

Caregivers expressed seeing their children engage in normal play activities was valuable for healthy development and as an indicator of recovery. One caregiver described,

“[She’s] had three strokes. So all the therapy and interaction helps with her rehabilitation process. So this is where she’s the happiest, playing with toys. [Her] Child Life [specialist] helps her stand up, play with toys, you know, get her interacting. And this is why I picked this photo, because it’s like, a strong part of her journey” (Adult Caregiver of 1 year-old patient).

Similarly, caregivers highlighted the importance of everyday experiences, from wearing regular clothes instead of hospital gowns to enjoying family movie nights, with one caregiver sharing,

“encouraging real clothes earlier in the process is nice. I kind of forgot about it this time, but it was so nice for her to like get back in her pants and her clothes when that was appropriate. And yeah, just like I know, it’s extra work for nurses to do things like this. And I’m comfortable pushing for that. But I know, you know, I’m a pretty experienced hospital parent. I know some other people might not be if this is their child’s first surgery or whatever. So nurses or you know, any staff going the extra mile to like set things up like this is really nice” (Adult Caregiver of 6 year-old patient).

While seeing their child engage in typical childhood behavior was an encouraging indicator of medical recovery, caregivers also recognized the importance of not rushing a return to “normal” and acknowledging that their child needed space to process their feelings, with one caregiver sharing, “I feel like we should let children feel… I think we forget about like, they’re still humans. If they feel like this, let them feel like this and just let them know we’re here. But it’s also valid, you know, whatever you’re feeling, it’s super validated.” (Adult Caregiver of 13 year-old patient).

Several caregivers voiced their desire for a normal childhood for their children while also expressing their interest in a conventional parenthood/caregiving for themselves despite being in a health care environment. They expressed desire to help care for their child through routine caregiving responsibilities, such as cleaning their child’s hair or changing their diaper. Caregivers described how they valued these moments as opportunities to fulfill their perceived role as a parent. One caregiver shared,

“This is the first time that I got to wash her hair after surgery. And I think this was like, over two weeks after surgery. We had washed it in the bed once, but I just was dying to get in there, like clean her head and to take care of her. It feels, you can feel really hands-off in medical situations, especially when she was so fragile. With the pacemaker situation I couldn’t get her out of bed. I couldn’t hold her, I couldn’t do all these things” (Adult Caregiver of 6 year-old patient).

Caregivers also voiced their desire to build memories with their child outside of the confines of their child’s illness. For example, one caregiver expressed how they wanted some of their photos to reflect their normal child-caregiver relationship rather than their child’s illness as they said:

“It was one of the first times holding him without the CPAP machine on, and you can see his little leg is so tiny. So I just wanted to be able to have a picture of the two of us together and unlike the other photos, this wasn’t maybe so much about like ‘Look what’s happening to him’ and ‘I want to be able to show other people’ as it was like I want to photo together with my son while I’m holding him, kind of checking each other out” (Adult Caregiver of 2 month-old patient).

#### Theme 4: joy, gratitude, and appreciating small things

Both patients and caregivers consistently expressed profound gratitude throughout their interviews. With many participants selecting photos with family as their most meaningful, they often conveyed gratitude for each other and for their families. When asked about a photograph of a drawing, one patient stated: “Not a whole lot of patients have parents that they can see almost every day. Some people do, but they’re not always here. So that’s why I have three hearts that have my dad and then me and my mom” (Pediatric Patient, 15 years old). Responding to what she thought others could learn from her photo, another patient advised to “always love your mom” (Pediatric Patient, 14 years old).

**Fig. 4:**
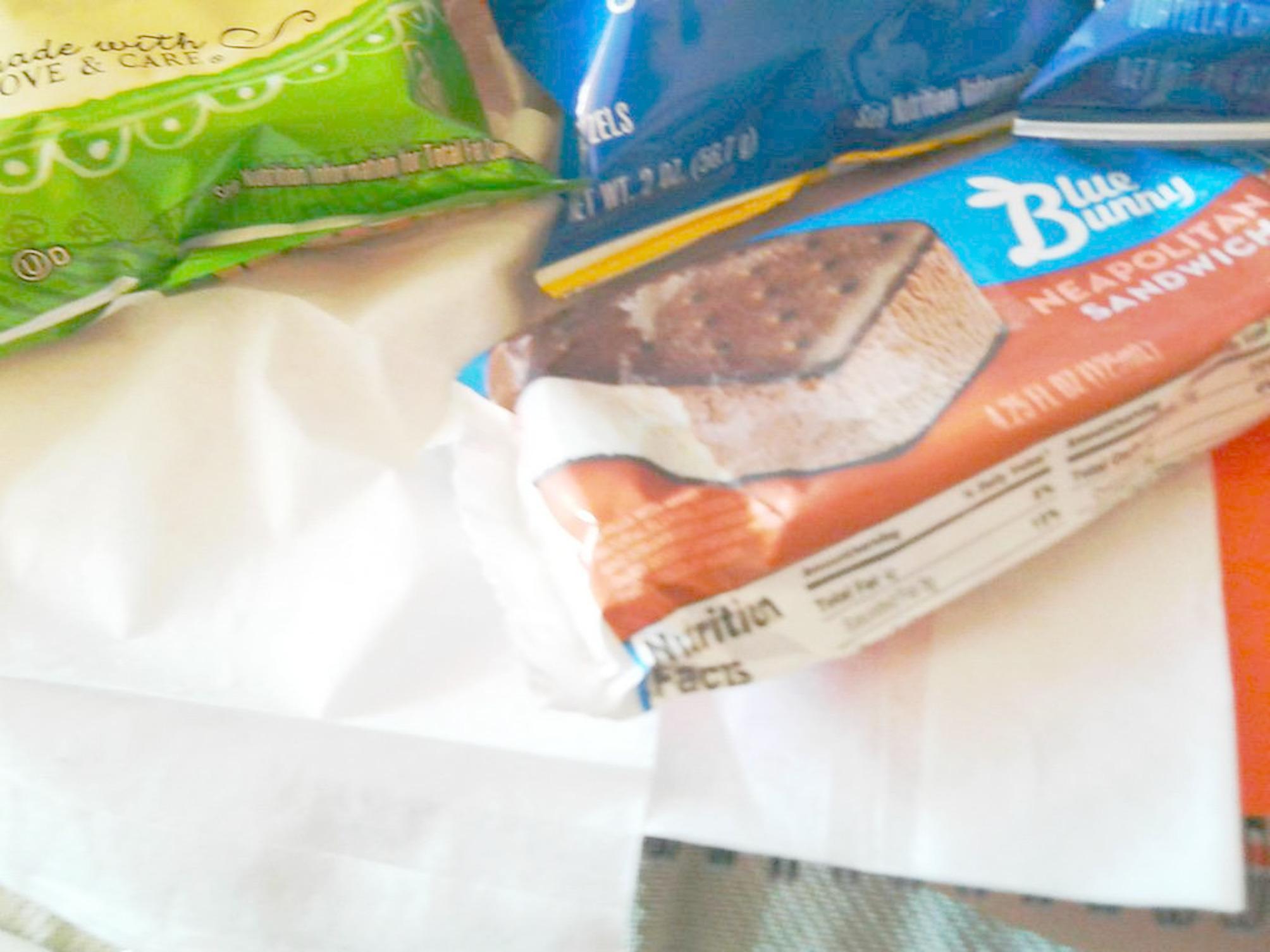
Photograph by patient of the snacks her family brought her when she could eat again

Patients and their families expressed gratitude for small, seemingly mundane things that provoked feelings of being loved and cared for, such as a stuffed animal or a home-cooked meal. One patient chose a seemingly ordinary photo of ice cream bars and chips that a family member brought her as her most meaningful photo:

“This photo was the first day that I could eat in a very long time. I was actually really happy because I was able to eat a lot of things that I love. The ice cream sandwich - it kind of made me sick because I ate chocolate on chocolate instead of the vanilla part because it’s cut in half. Then on top is the cooked kettle chips, the jalapeno. I really liked those. I used to eat those all the time when I was at home. And then yeah, this is my favorite” (Pediatric Patient, 15 years old) **(**Fig. 4**).**

These moments of gratitude in interviews were often followed by practical advice from patients, for example this patient concluded by saying “don’t lose faith, foodies” (Pediatric Patient, 15 years old).

Patient and caregivers also expressed appreciation for hospital staff and their clinical teams. One caregiver noted, “I think a lesson is like appreciation for the nurse who was like, yeah, if you want to hold your baby, you can hold your baby, and we’ll like, set it up” (Adult Caregiver of 2-month old patient).

While caregivers expressed comparable levels of gratitude during interviews, they uniquely expressed humor and gratitude amid challenging circumstances. One caregiver outlined how “even in the worst experience, you could always find some sort of joy and something that makes you smile” (Adult Caregiver of 5 year-old patient).

#### Theme 5: resilience and mental health

During our Photovoice-informed interviews, both children and caregivers expressed a deep awareness of how their experiences in the hospital significantly impacted their emotional wellbeing. For example, one pediatric patient noted, “I have a different life than other children because I have a heart condition, and it’s not the easiest” (Pediatric Patient, 11 years old). One caregiver shared, “I was pretty upset because I was really like he was so little he was just born. I was really afraid about what was happening” (Adult Caregiver of 2-month old patient).

**Fig. 5:**
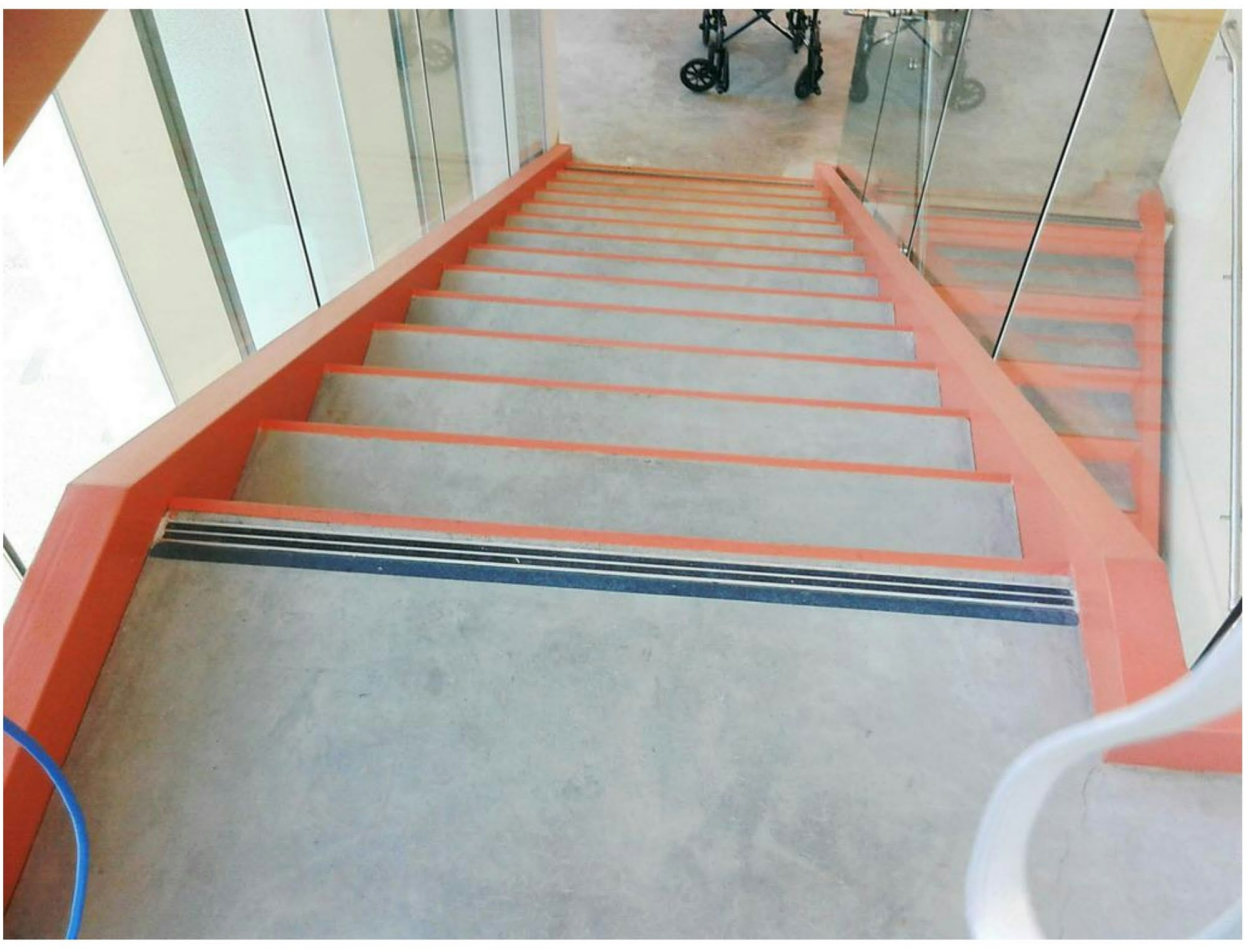
Photograph taken by pediatric patient shortly before discharge from the hospital

However, rather than seeing these images as simply documenting their medical and emotional challenges, both patient and caregiver participants discussed how the photos showcased their ability to overcome obstacles and be resilient. Upon reflection of her photo documenting her experience getting up out of her wheelchair and climbing the stairs for the first time (Fig. 5), one pediatric participant described the photo as a reminder that you can “always achieve your goals” and “you can do what you put your heart to” (Pediatric Patient, 11 years old). Reflecting on her family’s journey, one caregiver shared, “we bring color to our life, and we don’t let what’s happening, you know, put us in a dark place, because we keep the light shining, and the colors bright” (Adult Caregiver of 13 year-old patient).

Caregivers acknowledged that particular surgeries and procedures were painful and caused suffering for their children, but that images associated with these experiences represented hope for improved health in the future. Discussing a picture taken on the day of her child’s transplant, one caregiver noted,

“you know, for me, it’s like a happy day, you know, I see all the happy balloons in the back. You know, it’s there. I see. A happy day. But with her [my daughter] saying that she’s scared I, you know, I don’t see like any fear in the picture, per se. Colorful, like, I just feel like, like a big new beginning” (Adult Caregiver of 13 year-old patient).

**Fig. 6:**
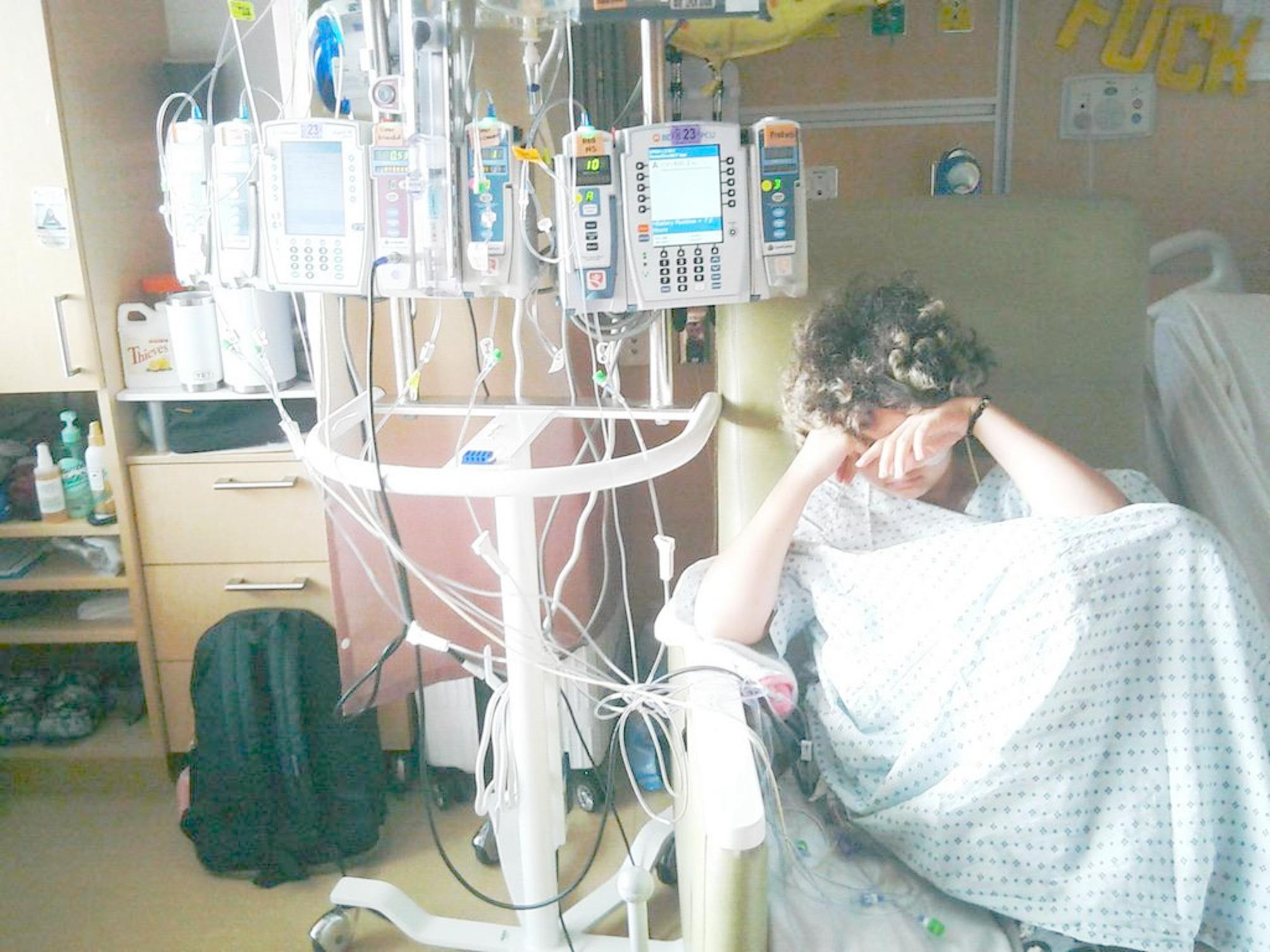
Photography by caregiver of her child receiving different treatments

Caregivers also noted that although they could see the long-term benefits of medical procedures, their child only experienced the immediate physical discomfort, creating difficult dynamics in which the same medical event in a photo represented necessary steps toward recovery for caregivers but suffering for the child. This caregiver provided further context, saying:

“When I see this photo, I see a child that is suffering, and just connected but disconnected at the same time, if that makes sense. Like she’s connected to all these things that are helping her stay alive, but you could see how disconnected she is from you know, what a child her age should be, you know? And, yeah, I see pain and suffering and just the reality of childhood cancer” (Adult Caregiver of 13 year-old patient) **(**Fig. 6**).**

Additionally, caregivers observed that, despite their child’s pain, these experiences fostered growth, maturity, and self-advocacy skills, creating images they hoped to share in the future as reminders of their child’s journey. One caregiver of a 6 year old patient shared:

“I’m really proud of her because she’s gotten really good at advocating for herself. And, you know, the last time she was three, so just a lot younger and not really able to, you know, ask nurses for what she wants. And she’s been so good at that this, like, you know, she’ll tell the EKG techs that she wants to take out the stickers every time they come in. Or like, as you just saw, she was like, I want to watch. I want to see. Don’t block me or don’t surprise me. You know, I’ve just been really proud of how she’s even just like from the first day of admission to now. Just how good she’s gotten advocating for herself” (Adult Caregiver of 6 year-old patient).

## Discussion

**Fig. 7:**
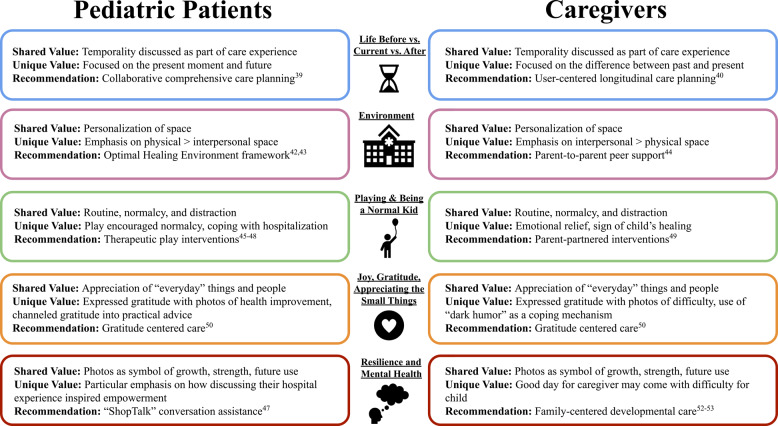
Care recommendation model derived from qualitative findings emphasizing patient and caregiver shared and unique perspectives

This study explored the experiences of pediatric patients and caregivers in the hospital environment through the unique lens of Photovoice-informed methodology. This novel methodological approach facilitates perspective sharing and expression through photography, yielding results that are particularly enriching and capture the nuances of hospitalization and illness. Other creative research practices have been employed in order to derive meaningful findings in pediatric hospital settings. For instance, written poetry is a medium used to give voice to pediatric patients [37], allowing patients to express complex descriptions related to space, time, self, and emotions. Unlike Photovoice, however, the visual aspect associated with care is lost. Hence, other studies have analyzed pediatric drawings to allow for the integration of both written word and environment; however, these can be limited by subjective interpretation of a child’s illustration when they are not paired with an interview/discussion [38]. Our findings revealed that the five identified themes captured both shared and differing values between pediatric patients and caregivers that together help inform specific care recommendations. Resultantly, we developed an exploratory care framework for use in similar pediatric care contexts, for instance, where patients have prolonged hospital stays and predominantly female caregivers (Fig. 7).

Both patients and caregivers acknowledged how quickly life could dramatically change. Interestingly, caregivers focused more on the stark differences between life before hospitalization versus their current situation, while emphasizing prior fears and their present gratitude. In contrast, pediatric patients seemed to focus on the present moment and the future, reflecting on how to use a newfound strength to move forward in their lives after or with illness. The temporality of patient and caregiver perspectives makes evident our recommendation for the incorporation of temporality into care discussions. Care is inherently longitudinal, and collaborative, comprehensive, and family unit-centered planning will help to support patients and their caregivers both during their hospitalization and after [39, 40].

Both patients and caregivers valued the personalization of hospital spaces and the outdoors. However, caregivers more often focused their reflections on the people around them and spatial dynamics, whereas patients focused more on physical objects, drawing symbolic insights from these objects and relating them to both hope and broader life. Similar themes were revealed in a study investigating parents’ perceived satisfaction with care, communication, and the hospital environment during a child’s pediatric ICU stay [41]. More than half of the parents included in that study were not satisfied with the noise level, time spent by nursing staff at the child’s bedside, or the methods used by the healthcare team to prepare for patient admissions. These were all factors highlighting social dynamics with others within the hospital environment. Our study, along with these others, therefore demonstrates that the environment is critical for pediatric patient outcomes and healing. Hence, a care recommendation derived from these findings emphasizes the use of the Optimal Healing Framework [42, 43], which outlines seven care components that support health, healing, and well-being. For instance, the framework recommends consideration of the physical care space in relation to techniques that foster healing through compassion, love, and awareness of interconnectivity. This could take the form of integrated parent-child creative practices, such as visual art projects, that could then be used to decorate the patient’s room, acting as a visual reminder of familial support and compassion. Furthermore, the integration of the Family Partner model has shown effectiveness in enhancing care for pediatric patients with complex care needs [44]. The Family Partner model provides interpersonal support networks for caregivers/parents navigating complex health circumstances through specialized training programing and professional support collaboration. Moreover, both groups highly valued regular routines and the feeling of “normalcy.” Both acknowledged the relief derived from the distractions of “normal life” outside of the hospital. However, differences in participant perspectives were also evident within this theme. Pediatric patients viewed play as a way to feel normal and cope with the hospitalization, whereas caregivers expressed a sense of emotional relief and perceived play as an indication of their child’s healing. Therefore, we recommend the incorporation of play-based interventions, which have been successfully integrated in other pediatric care settings [45–48]. Additionally, since caregivers expressed a desire to participate in their child’s everyday care activities, we recommend incorporating parent-partnered interventions, such as combined parent-child therapeutic board games or creative practices [49].

Both patient and caregiver participants emphasized their appreciation for their nearby support systems, their hospital staff, and even for seemingly mundane everyday tasks. However, patients more often focused on gratitude only when describing photos of their health journey improvement, whereas caregivers were more likely to express gratitude even during difficult moments. Finally, it was common for patients to also uniquely channel gratitude into practical advice to pass onto other patients. Expressions of gratitude are common and beneficial in healthcare settings for patients, caregivers, and practitioners, as a means to navigate the breadth of healthcare experiences that may be encountered within the hospital [50].

Our data also revealed the importance to participants of reclaiming their own narratives and framing their experiences through the lens of resilience. Patients particularly valued discussing their illness journey, reflecting on their progress, and exploring how to harness that strength moving forward. We therefore recommend creating structured opportunities for illness discussion, such as ‘ShopTalk,’ an innovative board game that facilitates emotional expression for children navigating illness [47]. Despite both groups describing the moments they captured as reminders of strength, growth, and progress, caregivers acknowledged that a moment that might be more positive to them (e.g., the day of transplant) might evoke more negative feeling in their child. This difference in resilience, emotions, and situational framing between caregiver-child dyads is well-documented in the literature [51], emphasizing that the caregiver and patient experience, while inextricably linked, are often misaligned. Hence, we recommend the incorporation of unit- or family-centered care given these critical experience gaps [52, 53]. We also recommend that future analyses focus on each caregiver-child dyad as a distinct unit, using Photovoice-informed methodologies to understand the different themes observed within each pair. Future studies may also consider conducting interviews both separately and jointly with caregiver-child dyads, which may both help researchers better understand differences in experiences between caregivers and children as well as facilitate development of an integrated narrative, which has shown to have been helpful in processing traumatic experiences [54].

Overall, both patient and caregiver narratives related to each of the themes underscored the utility of Photovoice as a method to both provoke reflection and capture moments that patients and families take with them beyond the timeline of the study. Of note, this study represents one component of a broader Photovoice initiative involving pediatric patients and their families. Only a subset of participants in the larger project took part in the formal research arm. The larger project has extended beyond data collection to include hospital-based and public gallery exhibitions, as well as wider efforts to share patient stories through creative and community-centered dissemination. These elements, though not detailed in this manuscript, reflect the broader purpose of Photovoice, not only as a research method, but as a tool for long-term engagement and impact within and beyond the hospital setting [11]. These insights will hopefully promote continued use of Photovoice among hospitalized pediatric patients and families.

There were some limitations associated with this study that must be acknowledged. For instance, while a heterogenous sampling method was used, the lack of male patient and caregiver participants may limit the generalizability of this work. Literature has explored the unique role of female caregivers in managing health-related tasks of their children when compared to their male counterparts [55]. It is well-established that female caregivers are more likely to report being the predominant caregiver responsible for addressing health-related tasks of their children when compared to males [55]. Hence, these findings emphasize the need for future studies that employ other recruitment strategies that would allow for the documentation of male caregivers and potential reasons for non-participation.

Similarly, the use of the purposive sampling strategy employed in this study may have led to additional recruitment biases, including a longer length of stay of participants when compared to the general pediatric patient population. Given children with longer hospital stays may be more likely to engage with Child Life programming in a longitudinal fashion and are therefore, are more likely to participate in a longitudinal study like this work, developing additional recruitment strategies for future research may help reduce biases associated with hospital stay length. It is possible that patients and caregivers in this study reflected a group that was medically able to partake in the study proceedings and interested in the creative format of Photovoice. Furthermore, our cohort mostly represented long-term inpatients and therefore, findings may not be applicable to acute care or emergent settings. Although this impacts generalizability, these limitations also provide a strong framework for future analyses, which could focus on these different medical domains. This initial analysis was also restricted to English-speaking patients, limiting the diversity of patients despite a more heterogenous sampling approach. Future research should consider non-English speaking patients, who likely have different perspectives navigating a predominantly English-based hospital environment. Additionally, we focused solely on photography as the creative medium through which participants could express their experiences artistically. Other mediums, such as visual arts and/or audio recordings could be incorporated to expand the breadth of expression and the patient population included in the study. Finally, the study questions did not focus on deriving specific recommendations or care experiences from patients. Hence, additional questions could be added to the interview guide to directly elicit actionable outcomes from interviews.

## Conclusions

We present key findings related to the pediatric and caregiver hospital experience through the use of Photovoice-informed methodology. Findings should be considered in the development of future care programming and planning to holistically improve the overall hospital experience for patients and their support networks. To improve care for pediatric patients and families during prolonged hospitalization, we offered several recommendations, including the use of Photovoice as a tool to empower patient families.

## Supplementary Information

Supplementary Material 1.

## Data Availability

The datasets used and/or analyzed during the current study are available from the corresponding author on reasonable request.

## Abbreviations

CPAP: Continuous positive airway pressure

## References

[1] O’Meara A, Akande M, Yagiela L, Hummel K, Whyte-Nesfield M, Michelson KN, et al. Family Outcomes After the Pediatric Intensive Care Unit: A Scoping Review. J Intensive Care Med. 2022;37(9):1179–98. 34919003 10.1177/08850666211056603

[2] Tang M, Chui PL, Chong MC, Liu X. Post-traumatic stress disorder in children after discharge from the pediatric intensive care unit: a scoping review. Eur Child Adolesc Psychiatry. 2025;34(2):483–96. 38916767 10.1007/s00787-024-02505-8

[3] Ko MSM, Poh PF, Heng KYC, Sultana R, Murphy B, Ng RWL, et al. Assessment of Long-term psychological outcomes after pediatric intensive care unit admission: A systematic review and Meta-analysis. JAMA Pediatr. 2022;176(3):e215767. 35040918 10.1001/jamapediatrics.2021.5767 PMC8767488

[4] Koch A, Kozhumam AS, Seeler E, Docherty SL, Brandon D. Multiple roles of parental caregivers of children with complex Life-Threatening conditions: A qualitative descriptive analysis. J Pediatr Nurs. 2021;61:67–74. 33780717 10.1016/j.pedn.2021.03.017 PMC8464614

[5] Stremler R, Haddad S, Pullenayegum E, Parshuram C. Psychological outcomes in parents of critically ill hospitalized children. J Pediatr Nurs. 2017;34:36–43. 28274664 10.1016/j.pedn.2017.01.012

[6] Lyu QY, Wong FK, You LM, Li XW. Perceived family impact during children’s hospitalization for treatment of acute lymphoblastic leukemia: a cross-sectional study. Cancer Nursing. 2020 Nov 1;43(6):489-97. 33084295 10.1097/NCC.0000000000000720

[7] Nelson LP, Lachman SE, Li SW, Gold JI. The effects of family functioning on the development of posttraumatic stress in children and their parents following admission to the PICU*. Pediatr Crit Care Med. 2019;20(4):e208–15. 30951005 10.1097/PCC.0000000000001894 PMC7518640

[8] Rennick JE, Knox AM, Treherne SC, Dryden-Palmer K, Stremler R, Chambers CT et al. Family members’ perceptions of their psychological responses one year following pediatric intensive care unit (PICU) hospitalization: qualitative findings from the caring intensively study. Front Pediatr. 2021;9:724155. 10.3389/fped.2021.724155 PMC8452961 34557460

[9] Colville G, Pierce CM. Post-traumatic stress trajectories of children and their parents over the year following intensive care discharge: A secondary analysis. Nurs Crit Care. 2024;29(4):830–4. 10.1111/nicc.13014 37994217

[10] Valenzuela JM, Vaughn LM, Crosby LE, Strong H, Kissling A, Mitchell MJ. Understanding the experiences of youth living with sickle cell disease: A photovoice pilot. Fam Community Health. 2013;36(2):97–108. 23455680 10.1097/FCH.0b013e318282b2f2 PMC4172332

[11] Catalani C, Minkler M. Photovoice: A Review of the Literature in Health and Public Health. Health Educ Behav. 37(3):424–51. 19797541 10.1177/1090198109342084

[12] Guillemin M, Drew S. Questions of process in participant-generated visual methodologies. Visual Stud. 2010;3(2):175–88.

[13] Drew S, Guillemin M. From photographs to findings: visual meaning-making and interpretive engagement in the analysis of participant-generated images. Visual Stud. 2014;29(1):54–67.

[14] Sobierajski F, Storey K, Bird M, Anthony S, Pol S, Pidborochynski T, et al. Use of photovoice to explore pediatric patients with hypertrophic cardiomyopathy and their parents’ perceptions of a Heart-Healthy lifestyle. JAHA. 2022;11(7):e023572. 35301849 10.1161/JAHA.121.023572 PMC9075448

[15] Graziano F, Toppino F, Vennettillo L, Abbate Daga G, Concas D, Mazzone G, et al. Promoting youth mental wellbeing: A photovoice project with adolescents and young adults in the hospital context. IJERPH. 2025;22(4):648. 40283869 10.3390/ijerph22040648 PMC12026857

[16] Sonsteng-Person M, García-Pérez J, Copeland V, Liévano-Karim L, Abrams D, Jarman B, et al. What I would do to take away your pain: A photovoice project conducted by mothers of children with medical complexity. Qual Health Res. 2023;33(3):204–19. 36704955 10.1177/10497323221146047

[17] Özdemir Koyu H, Algül G, Kilicarslan Törüner E. Realities and ideals: experiences and needs of pediatric oncology nurses in communication processes with children and their families at the end-of‐life period: A photovoice qualitative study. Nurs Health Sci. 2023;25(4):685–99. 37931643 10.1111/nhs.13062

[18] Ebrahimpour F, Mirlashari J, Hosseini ASS, Zarani F, Thorne S. Symbols of hope on pediatric oncology ward: children’s perspective using photovoice. J Pediatr Oncol Nurs. 2021;38(6):385–98. 34541954 10.1177/10434542211041934

[19] Barone S, Boss RD, Raisanen JC, Shepard J, Donohue PK. Our life at home: Photos from families inform discharge planning for medically complex children. Birth. 2020;47(3):278–89. 32808396 10.1111/birt.12499

[20] Bogetz J, Oslin E, Meissner E, Trowbridge A, Anderson J, Morris L, et al. A Photo-Narrative intervention for children with severe neurological impairment in the PICU. J Pain Symptom Manag. 2025;69(4):319–e33010. 10.1016/j.jpainsymman.2024.11.021 PMC11867884 39675393

[21] Wang C, Burris MA, Photovoice. Concept, Methodology, and Use for Participatory Needs Assessment. Health Educ Behav. 1997;24(3):369–87. 9158980 10.1177/109019819702400309

[22] Wang CC, Photovoice. A participatory action research strategy applied to women’s health. J Women’s Health. 1999;8(2):185–92. 10100132 10.1089/jwh.1999.8.185

[23] Balbale SN, Morris MA, LaVela SL. Using photovoice to explore patient perceptions of patient-centered care in the veterans affairs health care system. Patient. 2014;7(2):187–95. 24452963 10.1007/s40271-014-0044-5 PMC4419708

[24] Gorbenko K, Riggs A, Phlegar S, Koeppel B, Dubinsky M, Ungaro R, et al. Photovoice as a tool to improve patient - Provider Communicaiton in Inflammatory Bowel Disease Clniic: A pilot of feasibility study. Gastroenterology. 2021;160(3):S73–4. 10.1111/jep.13609 34382292

[25] Harper D. Talking about pictures: A case for photo elicitation. Visual Stud. 2002;17(1):13–26.

[26] Shaw PA. Photo-elicitation and photo-voice: using visual methodological tools to engage with younger children’s voices about inclusion in education. Int J Res Method Educ. 2021;44(4):337–51.

[27] Sutton-Brown CA, Photovoice. A Methodological Guide. Photogr Cult. 2014;7(2):169–85.

[28] Wang CC, Yi WK, Tao ZW, Carovano K. Photovoice as a participatory health promotion strategy. Health Promot Int. 1998;13(1):75–86.

[29] Foster-Fishman P, Nowell B, Deacon Z, Nievar MA, McCann P. Using methods that matter: the impact of Reflection, Dialogue, and voice. Am J Community Psychol. 2005;36(3–4):275–91. 16389500 10.1007/s10464-005-8626-y

[30] Drew SE, Duncan RE, Sawyer SM. Visual storytelling: A beneficial but challenging method for health research with young people. Qual Health Res. 2010;20(12):1677–88. 20729503 10.1177/1049732310377455

[31] Yi-Frazier JP, Cochrane K, Mitrovich C, Pascual M, Buscaino E, Eaton L, et al. Using Instagram as a modified application of photovoice for storytelling and sharing in adolescents with type 1 diabetes. Qual Health Res. 2015;25(10):1372–82. 25904674 10.1177/1049732315583282 PMC5565207

[32] Thomas DR. A General Inductive Approach for Analyzing Qualitative Evaluation Data. Am J Evaluation. 2006;27(2):237–46.

[33] Braun V, Clarke V. Using thematic analysis in psychology. Qualitative Res Psychol. 2006;3(2):77–101.

[34] Braun V, Clarke V. To saturate or not to saturate? Questioning data saturation as a useful concept for thematic analysis and sample-size rationales. Qualitative Res Sport Exerc Health. 2021;13(2):201–16.

[35] DeCuir-Gunby JT, Marshall PL, McCulloch AW. Developing and using a codebook for the analysis of interview data: an example from a professional development research project. Field Methods. 2011;23(2):136–55.

[36] Campbell JL, Quincy C, Osserman J, Pedersen OK. Coding In-depth semistructured interviews: problems of unitization and intercoder reliability and agreement. Sociol Methods Res. 2013;42(3):294–320.

[37] Wood D. Children’s experiences of hospitalization over time: an evaluation of using poetry and creative writing by children to assess their experiences of hospitalization. Patient Experience J. 2022;9(3):55–61.

[38] Buckle N, O’Neill A, Sweeney A, McNulty S, Bracken S, Awan A, et al. The use of Art as a creative research method to understand psychosocial care needs for children with rare diseases. Therapeutic Adv Rare Disease. 2024;5:26330040241265449.

[39] Adams S, Cohen E, Mahant S, Friedman JN, MacCulloch R, Nicholas DB. Exploring the usefulness of comprehensive care plans for children with medical complexity (CMC): a qualitative study. BMC Pediatr. 2013;13(1):10. 23331710 10.1186/1471-2431-13-10 PMC3570291

[40] Desai AD, Wang G, Wignall J, Kinard D, Singh V, Adams S, et al. User-centered design of a longitudinal care plan for children with medical complexity. J Am Med Inform Assoc. 2020;27(12):1860–70. 33043368 10.1093/jamia/ocaa193 PMC7727350

[41] Abuqamar M, Arabiat DH, Holmes S. Parents’ perceived satisfaction of care, communication and environment of the pediatric intensive care units at a tertiary children’s hospital. J Pediatr Nurs. 2016;31(3):e177–84. 26803562 10.1016/j.pedn.2015.12.009

[42] Ghazali R, Abbas MY. Natural environment in paediatric wards: status and implications. Procedia - Social Behav Sci. 2012;68:173–82.

[43] Iyendo TO, Uwajeh PC, Ikenna ES. The therapeutic impacts of environmental design interventions on wellness in clinical settings: A narrative review. Complement Ther Clin Pract. 2016;24:174–88. 27502819 10.1016/j.ctcp.2016.06.008

[44] Pollock MD, Ming D, Chung RJ, Maslow G. Parent-to-parent peer support for children and youth with special health care needs: Preliminary evaluation of a family partner program in a healthcare system. J Pediatr Nurs. 2022;66:6–14. 35597132 10.1016/j.pedn.2022.05.008

[45] Li WHC, Chung JOK, Ho KY, Kwok BMC. Play interventions to reduce anxiety and negative emotions in hospitalized children. BMC Pediatr. 2016;16(1):36. 26969158 10.1186/s12887-016-0570-5 PMC4787017

[46] Yogman M, Garner A, Hutchinson J, Hirsh-Pasek K, Golinkoff RM, Committee on Psychosocial, aspects of child and family health, et al. The Power of Play: A Pediatric Role in Enhancing Development in Young Children. Pediatrics. 2018;142(3):e20182058. 10.1542/peds.2018-2058 30126932

[47] Steele AC, Mullins LL, Mullins AJ, Muriel AC. Psychosocial Interventions and Therapeutic Support as a Standard of Care in Pediatric Oncology. Pediatric Blood & Cancer. 2015;62(S5). Available from: https://onlinelibrary.wiley.com/doi/10.1002/pbc.25701. Cited 11 Aug 2025. 10.1002/pbc.25701 26700919

[48] Liu LS, Inkpen KM, Pratt W. I’m Not Like My Friends: Understanding How Children with a Chronic Illness Use Technology to Maintain Normalcy. In: Proceedings of the 18th ACM Conference on Computer Supported Cooperative Work & Social Computing. Vancouver BC Canada: ACM; 2015:1527–39. Available from: 10.1145/2675133.2675201. Cited 11 Aug 2025.

[49] Power N, Franck L. Parent participation in the care of hospitalized children: a systematic review. J Adv Nurs 2008;62(6):622–41. 10.1111/j.1365-2648.2008.04643.x 18503645

[50] Day G, Robert G, Rafferty AM. Gratitude in health care: A Meta-narrative review. Qual Health Res. 2020;30(14):2303–15. 32924863 10.1177/1049732320951145 PMC7649920

[51] Mauritz PJ, Bolling M, Duipmans JC, Hagedoorn M. The relationship between quality of life and coping strategies of children with EB and their parents. Orphanet J Rare Dis. 2021;16(1):53. 33516244 10.1186/s13023-021-01702-x PMC7847038

[52] Hall SL, Cross J, Selix NW, Patterson C, Segre L, Chuffo-Siewert R, et al. Recommendations for enhancing psychosocial support of NICU parents through staff education and support. J Perinatol. 2015;35(S1):S29–36. 26597803 10.1038/jp.2015.147 PMC4660046

[53] Sedig LK, Spruit JL, Paul TK, Cousino MK, McCaffery H, Pituch K, et al. Supporting pediatric patients and their families at the end of life: perspectives from bereaved parents. Am J Hosp Palliat Care. 2020;37(12):1009–15. 32372700 10.1177/1049909120922973

[54] Kiser LJ, Baumgardner B, Dorado J. Who are we, but for the stories we tell: family stories and healing. Psychol Trauma: Theory Res Pract Policy. 2010;2(3):243–9. 10.1037/a0019893 PMC3010736 21197420

[55] Heffernan ME, Alfieri NL, Keese A, Bendelow AC, Casale M, Smith TL, et al. Differences in responsibility for child healthcare by parent gender: A cross-sectional study. Soc Sci Med. 2025;365:117576. 39647180 10.1016/j.socscimed.2024.117576

