# Supplementary material for: Reflections in focus: a qualitative Photovoice-informed study on pediatric patient and caregiver hospital experiences
Source: BMC Pediatr. 2026 Jan 9;26:53. doi: 10.1186/s12887-025-06281-5 (PMC12825289; doi:10.1186/s12887-025-06281-5)
Supplement: Supplementary file 1 — Supplementary Material 1. [file 12887_2025_6281_MOESM1_ESM.docx]

**Supplemental material 1:** Photo prompts given to pediatric patients and their caregivers.

For pediatric patients

1. Take a photo of something that captures your experience living with your condition
2. Take a photo that captures your experience going to the doctor
3. Take a photo of something that makes you feel hopeful
4. Take a photo of a challenge you face related to your health
5. Take a photo of something you find comforting at the doctor’s office/hospital
6. Take a photo of something you that makes you uncomfortable at the doctor’s office/hospital
7. Take a photo of something that changed your perspective on life with your condition
8. Take of photo of how you care for yourself when you’re in the hospital

For caregivers

1. Take a photo of what it’s like to care for your child in the hospital
2. Take a photo of how you help support your child in the hospital
3. Take a photo that captures your experience going to the doctor
4. Take a photo of something that makes you feel hopeful
5. Take a photo of a challenge you face in supporting your child at the hospital
6. Take a photo of something you find comforting at the doctor’s office/hospital
7. Take a photo of something you that makes you uncomfortable at the doctor’s office/hospital

**Supplemental Material 2:** Semi-structured interview guide used in Photovoice-informed study.

 Describe your photo (in as much detail as possible)?

- Probes:
  - Who is in this photo?
  - Where was this taken?
  - What machines are these in this photo?

What is happening in your picture?

- Probes:
  - Was this the first time this had happened?
  - How did this make you feel?
  - How has this experience changed throughout your experience with the healthcare system?

Why did you take a picture of this?

- Probes:
  - What were you feeling at that moment?
  - Would you have taken a picture of this before your healthcare experience?

What does this picture tell us about your life?

- Probes:
  - What does this picture tell us about the life of your family?
  - What makes this photo unique to your life instead of another child’s?

What lessons can we take from your photo to improve the experiences of other patients/caregivers**?**

- Probes:
  - What would you want other people to learn from your photo?
  - What would you want your family to learn from your photo?

The interview questions were adapted from the SHOWeD technique described by Wang et al. (1998), with modifications to simplify language for pediatric participants and tailor the prompts to the hospital context.

**Supplemental material 3:** Additional representative quotes

| Theme | Additional Quotations |
| --- | --- |
| 1. Life Before vs Currently  vs After | “100%, in this life, you know you’re like very good one day and a few minutes everything changes so drastically. It's more that you know everything changes in one second” (Adult    Caregiver of 5 year-old patient)    “we were back to doing diapers and things like that. And like, you know, having to be a little more hands on with like, helping her to the bathroom and things like that. So it did kind of seem like all of a sudden, I had like three really young kids again, because my other two are also in town” (Adult Caregiver of 6 year-old patient) |
| 2. Environment | “I took this [photo] because even though I'm not on a monitor all the time I would still have a picture of what my heart rate would be” (Pediatric Patient, 11 years old)    “we couldn't snuggle really because of the pacemaker. And because she had an [arterial] line. And [during] her last long stay, she didn't have all of that for a long time. So, I was able to like sleep in the bed with her. And so I felt a little more separated from it this time. So, it was really nice to like, get back in there and do something that made me feel like a Mom again” (Adult Caregiver of 6 year-old patient) |
| 3. Play and Being a Normal Kid | “we had our middle daughter come for a movie night. That's a tradition that we have at our house. We used to do movie nights every Saturday and have popcorn, and it's something you look forward to all week. [My husband] brought her in at like six o'clock, and [the staff] were like, ‘Oh, we usually don't let kids in like this late at night, but we'll let it slide.’ And like, Thank you, because we've been hyping them up for this for a while. So yeah, it was just really fun. Because this was, I think, only the second time that [her sister] had visited” (Adult Caregiver of 6 year-old patient)    “sometimes I feel like I've seen, like, parents not engaged so much in their children's activities throughout the day. They kind of like, let the nurses or other people do it. So I feel like it's very important to be active with your child in this type of environment and situation, because mental health is super important. I feel like if you're letting them or helping them, you know, get through it faster” (Adult Caregiver of 13 year-old) |
| 4. Joy, Gratitude and Appreciating Small Things | “I guess just having supportive staff is really nice and like staff that you feel like you can vent to or you know, just call for help. It's really nice to have those people” (Adult Caregiver of 6 year-old patient) |
| 5. Resilience and Mental Health | " I like [this photo] because it has my best stuffed animal in it and sometimes I like cuddling with it when I feel kinda scared” (Pediatric Patient, 5 years old).    “it's a dressing change from my Berlin. And it's just what I'm kind of here for to get a heart, I'm waiting on a heart and showing that I'm brave through these hard times” (Pediatric Patient, 12 years old) |
